# Characterization of Antigenic Relatedness Among GI Norovirus Genotypes Using Serum Samples From Norovirus-Infected Patients and Mouse Sera

**DOI:** 10.3389/fmicb.2020.607723

**Published:** 2020-12-08

**Authors:** Dongjie Xie, Junrui Chen, Jingrong Yu, Fuyu Pei, Mark Momoh Koroma, Lu Wang, Mengsi Qiu, Yuzhen Hou, Dexian Yu, Xu-Fu Zhang, Ying-Chun Dai

**Affiliations:** ^1^School of Traditional Chinese Medicine, Southern Medical University, Guangzhou, China; ^2^Guangdong Provincial Key Laboratory of Tropical Disease Research, Department of Epidemiology, School of Public Health, Southern Medical University, Guangzhou, China; ^3^Department of Pediatrics, Nanfang Hospital, Southern Medical University, Guangzhou, China; ^4^Guangzhou Military Command Center for Disease Control and Prevention, Guangzhou, China

**Keywords:** norovirus, immunotypes, GI genotypes, cross-reactivity, cross-blockade, HBGA, vaccine strategy

## Abstract

Characterizing diversity and the antigenic relatedness of norovirus remains a primary focus in understanding its biological properties and vaccine designs. The precise antigenic and serological features of GI genotypes have not been studied. The study represented an investigation on a gastroenteritis outbreak related to GI.3 norovirus and the three most detected GI genotypes, GI.2 (belonging to immunotype B), GI.3 and GI.9 (belonging to immunotype C), were selected to characterize their phylogenetic relationship, HBGA binding profiles and antigenic relatedness within (intra-immunotype), and between (inter-immunotypes) genotypes using mouse sera and patient’s serum samples from the GI.3 related outbreak. Wide HBGA binding profiles and evolution of binding affinity were observed in the three GI genotypes studied. A low specific blockade antibody to GI.3 in the population generated the pool of susceptible individuals and supported virus spread in the outbreak. We found strong blockade immune response in homologous strains, moderate intra-immunotype blockade but weak inter-immunotypes blockade in humans following GI.3 norovirus infections. These findings further support the immunotypes grouping and will be valuable for optimizing the design of norovirus vaccine.

## Introduction

Noroviruses (NoVs) are the most common global viral cause of acute gastroenteritis (AGE) affecting all age groups (Ahmed et al., 2014; Van Beek et al., 2018). NoVs account for a fifth (18%) of all AGE cases with annual deaths of 70,000–200,000 (Karst et al., 2015; Bányai et al., 2018). Despite the estimated global economic impact of more than $64 billion per year (Hall et al., 2013; Atmar et al., 2018), vaccine availability for prevention is still lacking. NoV related AGE usually have a short incubation period of 10–50 h, with primary symptoms of either vomiting, watery diarrhea and abundant viral shedding after infection (Atmar et al., 2018).

NoVs are non-enveloped, single-stranded positive-sense RNA viruses with a genome length of 7.5–7.7 kb and belong to the *Caliciviridae* family (Nilsson et al., 2003). The genomes are organized into three open reading frames (ORFs) that encode for both structural and non-structural proteins (Karst et al., 2014; Cannon et al., 2017). Of these, ORF2 encode the major structural protein, the capsid protein (VP1). VP1 consists of a highly conserved shell (S) domain and a protruding (P) domain, which is response for receptor binding and neutralizing antibodies production (Bányai et al., 2018). The P proteins is formed *in vitro* that retain the immunogenic and the receptor binding function (Tan et al., 2008).

Depending on the amino acid sequence of VP1, NoVs can be classified into ten genogroups (GI-GX) (Chhabra et al., 2019). Of these, genogroup I (GI) and genogroup II (GII) account for most human infections, which can be divided into at least nine (GI.1-GI.9) and 27 (GII.1-GII.27) genotypes, respectively. Though GII variants show a higher prevalence in humans with multiple NoV genotypes co-circulating (Ramani et al., 2014; Saito et al., 2014), an increase of GI NoV activity from 7.8% to 37.3% was observed in the last decade (Hasing et al., 2013). The distribution of GI variants as the most predominant strain was reported in sub-Saharan Africa with GI.7 (33%), GI.3 (21%), and GI.5 (17%) from 1993 to 2015 (Munjita, 2015). Moreover, a study investigating the prevalence and characteristics of asymptomatic NoV infection suggested GI.9, GI.2, and GI.3 accounted for 39.7% asymptomatic infection among people living around oyster farms in south China (Wang et al., 2018). A surveillance analysis of AGE cluster in Taiwan revealed 16.5% NoV positive for GI of which GI.3 was the most predominant genotypes accounting for 36.8% thus suggested a close monitor of GI.3 prevalence (Chiu et al., 2020).

NoVs use glycans of the ABH and Lewis histo-blood group antigen (HBGA) family for attachment to their target cells (Tan and Jiang, 2014). Significant genotypic and phenotypic diversity of HBGA expression exists between different human populations (Nordgren and Svensson, 2019). Several studies have demonstrated that NoV strains differ in their ability to bind HBGAs and have associated NoV susceptibility (Huang et al., 2005; Marionneau et al., 2005; Shirato et al., 2008).

Previous volunteer challenge studies have indicated a lack of protection between GI and GII (Yang et al., 2010; Swanstrom et al., 2014). Diverse NoV genotypes have been shown to re-infect children within the first 5 years of age and resulted in immunotypes or serotypes been proposed for rational NoV vaccine design (Malm et al., 2014). The known nine GI genotypes have been grouped into three immunotypes (A–C) (Parra et al., 2017). Reactivity or blockade between immunotypes is termed inter-immunotypes while reactivity or blockade within an immunotype is termed intra-immunotype. Currently, NoV evolution, host susceptibility, and serological functions primarily focus on GII genotypes (White, 2014; Currier et al., 2015; Sharma et al., 2017). However, studies on the serological correlation between GI genotypes-induced gastroenteritis protection and antigenic relatedness are limited.

In this study, the three most detected GI genotypes, GI.2 (immunotype B), GI.3 and GI.9 (immunotype C), were selected to characterize their phylogenetic relationship, HBGA binding profiles and antigenic relatedness within (intra-immunotype) and between (inter-immunotype) genotypes using mouse sera and patient’s serum samples from NoV GI.3 related outbreak.

## Materials and Methods

### Outbreak Investigation and Samples Collection

On 10th July 2018, an AGE outbreak occurred in Guigang City, Guangxi Province, China, with 429 individuals reported illness (Xing-Chao et al., 2019). A subset of the study samples from the large outbreak was obtained in a military setting using a questionnaire (Xing-Chao et al., 2019). Symptomatic cases were defined as individuals who experienced symptoms of at least one either episode of vomiting or diarrhea or both starting from the onset date. Asymptomatic cases were defined as those whose stool specimen or rectal swab was positive for NoVs, but who did not experience symptoms of vomiting or diarrhea (Zhang et al., 2015).

A total of 42 stool swab samples were collected, 10 out of 15 symptomatic subjects and the rest from asymptomatic individuals. Thirty-nine paired serum samples were collected, among which 15 from symptomatic individuals at both acute phase (within a week after onset date) and convalescent-phase (approximately 5 weeks after the onset date), and the remainders from asymptomatic individuals in the same setting at a parallel time.

### NoV Genotyping, Sequences and Amino Acid Analysis of P Domain of Different GI NoV

The viral RNA extracted from rectal swabs was detected by one-step RT-PCR assay using GI and GII primers (Wolf et al., 2007). Further genotyping was performed with region-C-specific primers using one-step RT-PCR (Qiagen, CA, United States) (Kojima et al., 2002). The positive PCR products were sequenced and determined by NoV genotyping tool (Kroneman et al., 2011).

The P domain of the outbreak (GI.3) and GI.9 strains were amplified using primers GI.3-F: 5′-ATCGCGGATCCCAAA AGACT-3′, GI.3-R: 5′-GCATGCGGCCGCTTAGCAAAAGCAA TCGCCAC GGCAATCGCATATAC-3′, GI.9-F: 5′-TCGCGG ATCCCAAAAGACC-3′ and GI.9-R: 5′-GCATGCGGCC GCTTAGCAAAAGCAATCGCCACGGCAATCGCATACTCT-3′, respectively. The reverse primer was linked by a cysteine-containing peptide (P-CDCRGDCFC) to C-terminus, as described previously (Tan and Jiang, 2005). The P domain encoding sequences were cloned into T-vectors (Thermo Fisher, United States). The resulting variant fragments were GI.3 (the outbreak strain), GI.9 (extracted from stool sample provided by Guangdong CDC) (Wang et al., 2018) and GI.2 (accession number KF306212).

The P domain sequences of representative selected strains, together with the three strains in our study were aligned with reference strains using DNAstar 7.1, and phylogenetic trees were constructed using the neighbor-joining method (MEGA 6.0). To analyze the evolution of HBGA-binding interfaces and the surrounding regions, amino acid analysis and comparison of these regions were also conducted.

### Expression of P Proteins

The cDNAs encoding the capsid P domain were cloned into the expression vector pGEX-4T-1 (Amersham Biosciences, Piscataway, NJ, United States) between *Bam*HI and *Not*I sites. After sequence confirmation, P proteins were expressed in *Escherichia coli* following previously described procedures (Tan et al., 2008). Briefly, The BL21 cultures were initiated by 0.5 mM IPTG (isopropyl-β-D-thiogalactopyranoside) at a temperature of 22°C overnight. The recombinant P domain-GST fusion proteins were purified using Glutathione Sepharose 4 Fast Flow resin (GE Healthcare Life Sciences, NJ, United States). The GST was removed from the P proteins by thrombin (GE Healthcare Life Sciences, Princeton, NJ, United States) cleavage on beads at 22°C overnight.

### Generation of Mouse Antiserum

Seven- to 8-week-old female BALB/c mice (*n* = 4 for each group) were subcutaneously immunized four times at two-week intervals with P proteins plus adjuvant at a volume ratio of 1:1, respectively (Invitrogen Life Technologies Carlsbad, CA, United States). An initial dose of 60 μg P proteins of each GI.2, GI.3 or GI.9 with complete Freund’s adjuvant was used, followed by three booster doses of 30 μg with incomplete Freund’s adjuvant. At the same time, the control groups received phosphate-buffered saline (PBS) with adjuvant. Blood serum samples were collected from the mice and pooled for each group. Animal experimental protocol (No. SCXK 2016-0041) was approved by the Animal Care and Ethics Committee of Southern Medical University.

### HBGA Binding Profiles of GI.2, GI.3, and GI.9 NoV

A panel of saliva samples provided by volunteers with well-defined HBGA phenotypes were tested in a saliva-based HBGA-P protein-binding assay as described previously (Zhang et al., 2015). Saliva samples were boiled for 10 min at a diluted concentration of 1:1,000, then coated onto 96-well plates (Costar, Corning, CN) overnight at 4°C. After blocked with 5% non-fat milk in PBS. 5 μg/ml of P proteins was added and incubated for 1 h at 37°C. Our in-house made mice anti-GI.2, GI.3, and GI.9 NoV diluted at 1:3,000 sera were then added, followed by HRP-conjugated goat anti-mice IgG at 1:5,000. Signals were developed using a tetramethylbenzidine (TMB) substrate kit. The cut off positive signal of Optical Density 450 (OD_450_) was set at 0.2, negative control mean OD plus three times of standard deviation.

### IgG Antibody Assay

GI.2, GI.3, and GI.9 specific IgG antibody in immunized mouse serum and human serum samples were analyzed using enzyme-linked immunosorbent assay (ELISA). Plates were coated with 0.5 μg/ml diluted P protein of GI.2, GI.3 or GI.9 overnight at 4°C. Mouse or human serum were added at twofold serial dilution starting at 1:500. Then horseradish peroxidase (HRP)-conjugated goat anti-human IgG or goat anti-mice IgG was used to detect bound NoV antibodies. The positive cut-off signal was set at 0.2 and a titer of <500 was assigned to a value of 250. Seroconversion was defined as ≥a fourfold increase in titer of the convalescent-phase serum sample compared with that of the acute-phase serum sample.

### Serum Antibody Blockade Assay

Based on results of the above HBGA binding profiles testing, candidate saliva samples, Volunteer 23 (V23, phenotype: A) to GI.2 and GI.3, Volunteer 36 (V36, phenotype: O) to GI.9, demonstrating strong binding signals, were selected for HBGA blockade assay. The HBGA binding assay consisted of an additional blockade step of P proteins. The P proteins at 0.5 μg/ml were pre-incubated in twofold serial dilution (1:25 to 1:3,200) with mice antisera or human sera at 37°C and then applied to the coated saliva.

Additionally, the blockade assay with antisera from mice and the bound P proteins were detected using our in-house made guinea pig anti-NoV (immunized with mix GI.2, GI.3, and GI.9 P proteins) sera at 1:2,000, followed by HRP-conjugated goat anti-guinea pig IgG (ICN, Aurora, OH, United States). The P proteins without preincubation with a serum sample were used as a positive control that showed optical density (OD) values within the range of 1.0 ± 0.3 (Dai et al., 2017). The 50% blocking titer (BT_50_), was defined as the maximal dilution (in folds) of a serum sample that showed at least 50% blockade in OD compared with the positive control. A value of 12.5 was assigned to a serum sample with a BT_50_ < 25.

### Statistical Analyses

Spearman’s rank correlation was conducted between genotype specific IgG and serum blockade titers. The correlation coefficients of genotype specific IgG antibody titers, blocking titers and increase folds were used to examine the discrepancies between different strains. The Mann–Whitney *U* test was used to compare increase folds of GI.3 between the symptomatic group and the asymptomatic group. Statistical analyses were conducted with IBM SPSS software version 22.0 (SPSS Inc., Chicago, IL, United States) and the statistical significance was defined as *P* < 0.05.

## Results

### Gastroenteritis Outbreak Caused by GI.3 NoV

There were 93 male soldiers in the military camp, 15 of whom reported sick with AGE on 11th July 2018. All ten samples collected from symptomatic individuals and 6 of the 32 samples (18.5%) from asymptomatic individuals were positive for GI.3 NoV. Of note, one of the symptomatic subjects (GG14) was co-infected with both GI.3 and GII.17 NoV. The basic demographics, symptoms and stool test results of these 15 symptomatic and 6 asymptomatic individuals are shown in Table 1.

**TABLE 1:** Basic demographics, symptoms, and rectal swabs of study population.

Groups	Subject	Age (years)	Patients within 24 h with:				Rectal swabs test results for norovirus
			Vomiting	Diarrhea	Fever	Other symptoms^a^	Genotype
Symptomatic cases *N* = 15	GG01	21		√	√		GI.3
	GG02	25	√	√			GI.3
	GG03	22		√	√		
	GG04	26		√	√		GI.3
	GG05	23		√			
	GG06	24		√	√		GI.3
	GG07	31	√	√			GI.3
	GG08	22				√	
	GG09	26	√	√			GI.3
	GG10	19		√			
	GG11	23	√				GI.3
	GG12	25	√	√	√		GI.3
	GG13	27				√	
	GG14	23	√	√			GI.3/GII.17
	GG15	22	√	√			GI.3
Asymptomatic controls *N* = 6	GG19	20					GI.3
	GG24	24					GI.3
	GG31	26					GI.3
	GG37	23					GI.3
	GG38	29					GI.3
	GG40	22					GI.3

*^*a*^Abdominal pain, muscular soreness, and tiredness.*

### Phylogenetic Analysis of P Domain Sequences

**FIGURE 1:**
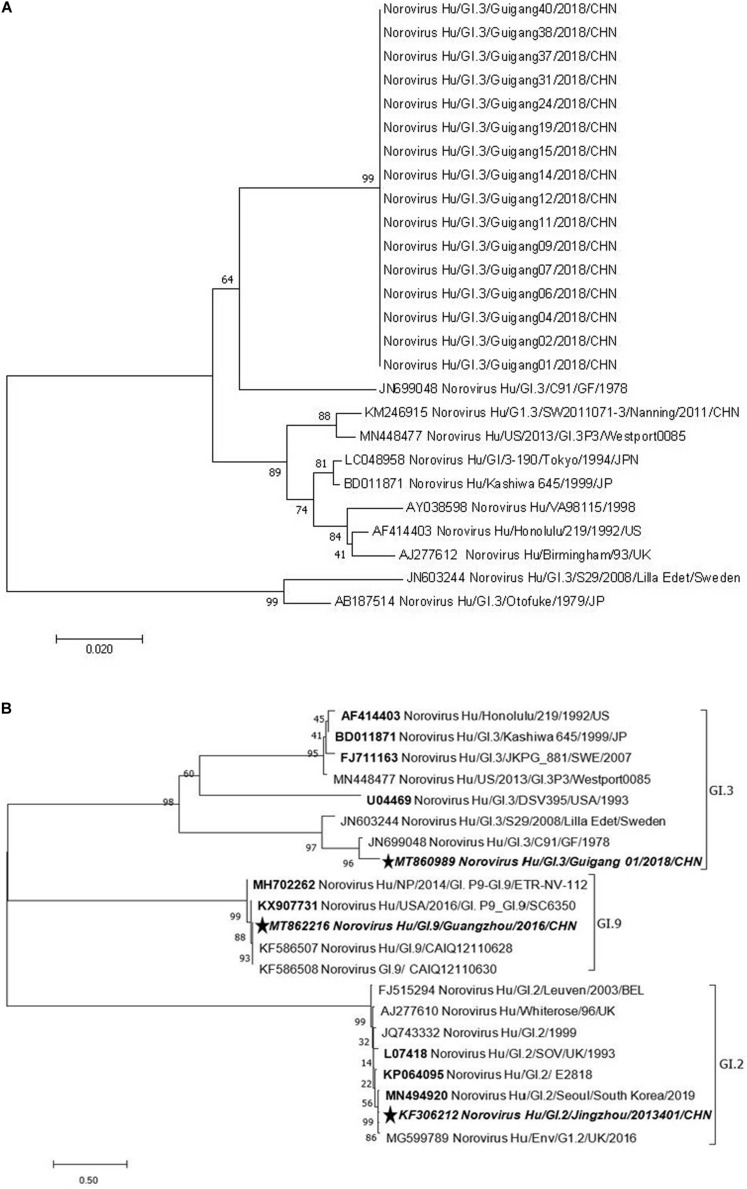
Phylogenetic tree analyses based on nucleotide sequences encoding capsid NS terminus of the outbreak strains (330 bp fragment) **(A)** and P domain **(B)**. The sequences used to express P proteins in this study were marked with pentagram. The bootstrap values generated from 1,000 replicates are shown at each node.

A phylogenetic analysis of NS terminus in capsid protein was performed. Sixteen sequences, 10 from the symptomatic and 6 from the asymptomatic displayed 99.0–100% nucleotide identity, indicating a GI.3 NoV-related outbreak (Figure 1A). Further phylogenetic analysis of P domain sequence of GG01 isolated from the outbreak showed a closer homology with GI.9 (64.5%) than the GI.2 strain (58.7%) (Figure 1B). The outbreak virus and GI.9 virus were designated as Norovirus Hu/GI.3/Guigang 01/2018/CHN and Norovirus Hu/GI.9/Guangdong/2016/CHN, and the GenBank accession numbers as MT860989 and MT862216, respectively.

### The HBGA-Binding Interfaces of GI.2, GI.3 and GI.9 NoV

**FIGURE 2:**
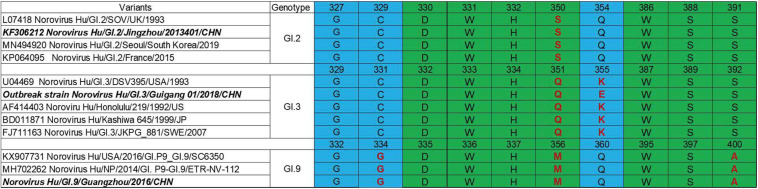
Comparisons of HBGA binding interfaces in green and the surrounding regions in blue of GI.2, GI.3, and GI.9 noroviruses. Substitutions of amino acid are shown in red.

P domain (963 bp) of GI.2 corresponded to VP1-encoding Gene nucleotides (nt.) 679–1641, GI.3 (957 bp) to nt. 679–1635 and GI.9 (969 bp) to nt. 685–1653. Sequence alignment revealed evolutionarily conserved amino acids in HBGA-binding interfaces and the surrounding regions (Figure 2) with some variations. Residue differences were observed in region 1 (S/−350, Q/−351, and M/−356) respectively, in the HBGA-binding interfaces of GI.2, GI.3, and GI.9 NoVs. Additionally, one mutation of A/−400 was observed in region 2 of binding interfaces of GI.9 compared to GI.2 and GI.3. Lastly, the surrounding regions of GI.9 and GI.3 also revealed two residual differences in G/−334 and K/−355.

### The HBGA Binding Properties of GI.2, GI.3 and GI.9 NoV

**FIGURE 3:**
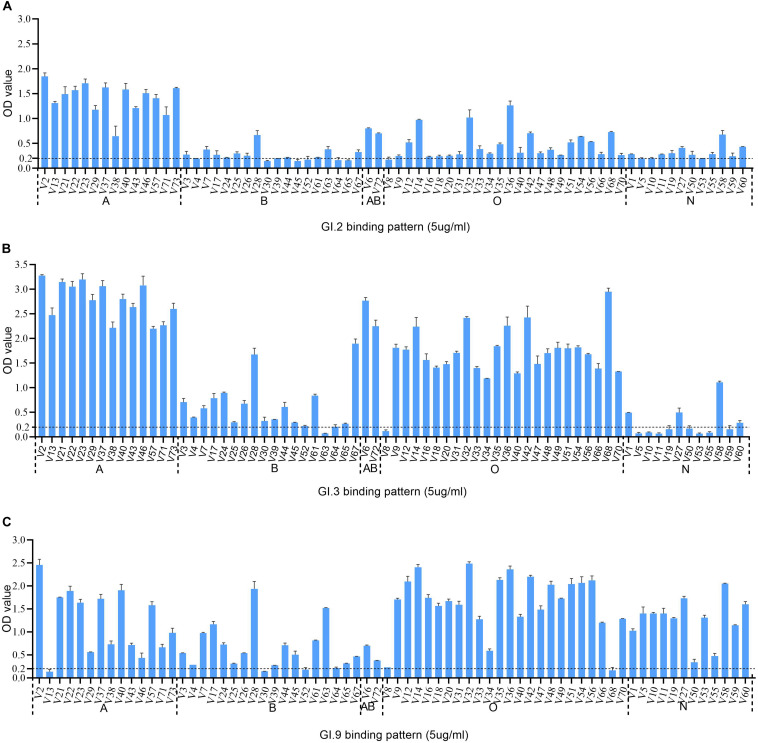
HBGA binding profiles of GI.2 **(A)**, GI.3 **(B)**, and GI.9 **(C)** P proteins to saliva samples. A panel of saliva samples with confirmed phenotypes were used for HBGA binding profiles (Zhang et al., 2015). The binding signals are determined by optical density (Y axis). At the same time, the well-characterized saliva samples of volunteers (V as abbreviated for volunteers) are shown in X-axis. Various A, B, AB, O, and N stand for blood type A, B, AB, O, and non-secretor, respectively.

The HBGA binding assay showed that all the three GI NoV could bind to A, B, AB, and O secretors and non-secretors with varying affinities. The binding affinity of GI.2 was highest for type A secretor (mean OD: 1.41 ± 0.31), as shown in Figure 3A. While the outbreak strain (GI.3), bounded more efficiently to saliva type A (mean OD: 2.77 ± 0.38), AB (mean OD: 2.51 ± 0.37), and O secretors (mean OD: 1.70 ± 0.54) than that of type B (mean OD: 0.62 ± 0.49) secretors and non-secretor (mean OD: 0.27 ± 0.31) shown in Figure 3B. However, GI.9 had good binding activities for all type A (mean OD: 1.23 ± 0.70), B (mean OD: 0.65 ± 0.49), AB (mean OD: 0.54 ± 0.23), and O (mean OD:1.65 ± 0.63) secretors and non-secretors (mean OD: 1.27 ± 0.48) (Figure 3C), but with variations in OD values (Figure 3C).

### Inter- and Intra-Immunotypes Cross-Reactivity and Blockade Using Mouse Hyperimmune Anti-NoV Sera

**FIGURE 4:**
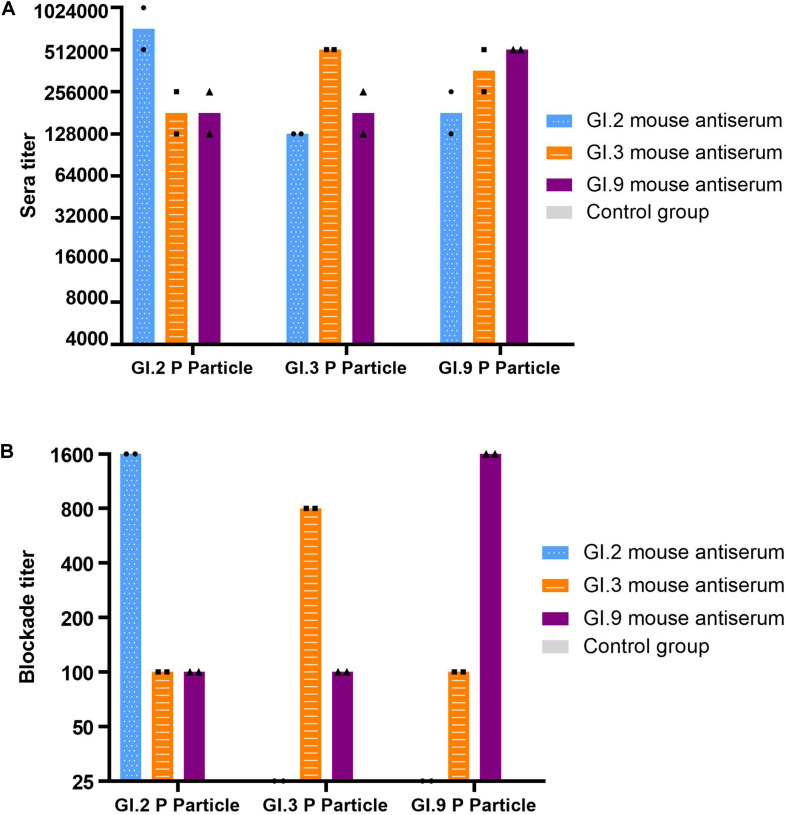
IgG titers **(A)** and blockade titers **(B)** to GI.2, GI.3, and GI.9 of the immunized mouse sera. The y-axis indicates the specific antibody titer **(A)** and blockade titer **(B)**. The blue, orange, and purple bars indicate the mouse antiserum of GI.2, GI.3, and GI.9, respectively.

The specific IgG titers against homologous strains were the highest (Figure 4A). The IgG titers against heterologous intra-immunotype C strains (GI.3 and GI.9) were twofold to fourfold lower than that against the homologous strain. In comparison, the IgG titers against heterologous inter-immunotypes (immunotype B against immunotype C) were twofold to eightfold lower. NoV-specific IgG was not developed in the control group of mice sera (Figure 4A).

Similar to the specific IgG, the most potent blocking abilities were observed against homologous strains, with titer ranging from 800 to 1,600. Serum of GI.3 and GI.9 showed low blocking effect against to heterologous strains at a titer of 100. However, the serum of GI.2 failed to exert the blocking effect against GI.3 and GI.9 (Figure 4B).

### Specific and Cross-Reactive IgG Antibody in Humans Following GI.3 NoV Infections

**FIGURE 5:**
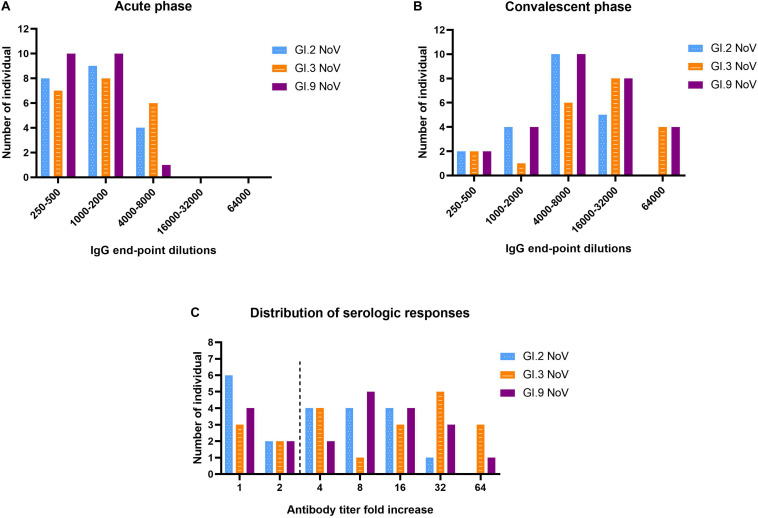
Serologic responses of IgG titers to GI.2, GI.3, and GI.9 of the paired human sera collected from the GI.3-related outbreak. Distribution of IgG titers at the acute phase **(A)**, convalescent phase **(B)**, and serological fold rises **(C)** among 21 individuals. The blue, orange, and purple bars indicate serologic responses to GI.2, GI.3, and GI.9, respectively.

The majority of individuals (66.7%, 14/21) possessed IgG antibody titers between 1,000 and 8,000 against GI.3 (Figure 5A). Following NoV infection with the GI.3, sera in convalescent phase exhibited the strongest IgG response against homotypic GI.3 (Figure 5B), and 16 out of 21 paired sera (76.2%) exhibited seroconversion (4–64-fold increase) (Figure 5C). Heterotypic serum antibody titers against GI.9 and GI.2 were also induced. Fifteen of the 21 (71.4%) exhibited seroconversion (4–64-fold) against GI.9 and 13 out of 21 (61.9%) exhibited seroconversion (4–32-fold) in GI.2 (Figures 5B,C). There was no statistical difference for the specific IgG titer, the seroconversion rate and increased folds among the three strains. Positive correlations were detected between the increase folds of GI.3 and GI.9 (*r* = 0.931, *P* < 0.001) and between the increase folds of GI.3 and GI.2 (*r* = 0.914, *P* < 0.001). Of note, there was no significant difference of increase folds to GI.3 (*P* = 0.179) between the symptomatic patients and asymptomatic patients.

### Strong Homologous Blockade, Moderate Intra-Immunotype Blockade, but Weak Inter-Immunotypes Blockade in Humans Following GI.3 NoV Infection

**FIGURE 6:**
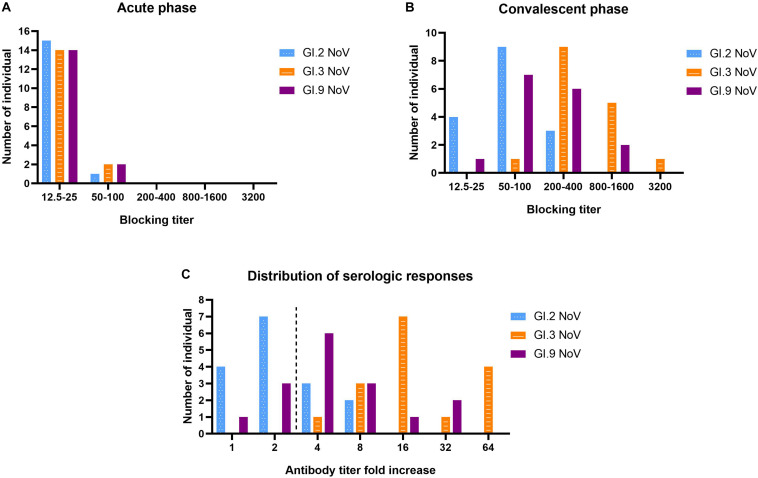
Serologic responses of blockade titers to GI.2, GI.3, and GI.9 of the paired human sera collected from the GI.3-related outbreak. Distribution of blockade titers at the acute phase **(A)**, convalescent phase **(B)**, and serological fold rises **(C)** among 16 individuals with IgG seroconversion. The blue, orange, and purple bars indicate serologic responses to GI.2, GI.3, and GI.9, respectively.

Our results revealed that serum samples at acute-phase exhibited very minimal blockade, with only 9.52% (2 out of 21) showing blockade antibody titers at 50 against the outbreak strain GI.3 (Figure 6A), which indicated a low herd protective immunity at the beginning of the outbreak. However, sera at convalescent-phase displayed a noticeable rise in blocking titers (Figure 6B). Among the 16 individuals with seroconversion, blockade titers exhibited 4–64-fold increase against the GI.3 genotype, and 75% (12/16) showed a comparable 4–32-fold increase against GI.9 (Figure 6C). In contrast, only 31.25% (5/16) showed fourfold to eightfold increase against GI.2 variant (Figure 6C). Of the three, GI.3 genotype caused the most robust blocking antibody response to homologous strain; and induced a more robust response to GI.9 than that to GI.2. The results revealed strong blockade in homologous strain (GI.3), moderate intra-immunotypes (GI.9) blockade, but weak inter-immunotypes (GI.2) blockade in humans following GI.3 NoV infection.

There was also no significant correlation between specific IgG titer and BT_50_ of GI.3 at both the acute phase (*r* = 0.232; *P* = 0.388) and convalesce phase (*r* = 0.247; *P* = 0.357). Further analysis exhibited a positive correlation (*r* = 0.5323, *P* = 0.037) between the increase folds of BT_50_ of GI.3 and GI.9, In contrast, no significant correlation was exhibited between the increase folds of BT_50_ of GI.3 and GI.2 (*r* = 0.263, *P* = 0.324). Similarly, there was no significant difference in increase folds of blocking titers to GI.3 between the symptomatic patients and asymptomatic patients (*P* = 0.775).

## Discussion

Characterizing the diversity and the antigenic relatedness of NoV remains a primary focus in understanding its biological properties and vaccine designs. Due to the diverse antigenic nature of NoVs, current bivalent or multivalent vaccine design is targeted to have a minimum requirement of GI and GII (Atmar and Estes, 2012; Debbink et al., 2014). While several studies have targeted antigenic diversity of predominant GII strains (Parra et al., 2012; Dai et al., 2017), only a few studies involved GI prevalent strains in natural environmental infections (Farkas et al., 2003). The precise serological and antigenic features of GI genotypes have not been studied *in vitro*. Also, the risk related to GI NoVs infection requires further understanding, which can be evaluated by the susceptible host range, the level of pre-existing immunity, and cross-reactivity with other circulating stains.

During July 10–14, 2018, a large GI-NoV related outbreak was reported in Guigang City due to contaminated peripheral water (Xing-Chao et al., 2019), which was consistent with most outbreaks of GI strains associated with waterborne and foodborne transmissions in a systematic review (Verhoef et al., 2015). A subset of individuals was recruited in a military setting, with the same centralized source of water supply mainly contaminated with GI.3 NoVs (Xing-Chao et al., 2019). In this study, two NoV strains belonging to immunotype C (GI.3 and GI.9) and one strain belonging to immunotype B (GI.2) (Parra et al., 2017) were selected, aiming to analyze phylogenetic relationships and HBGA binding spectra of 3 different GI genotypes to evaluate pre-existing GI.3-specific immunity at the beginning of the outbreak; and to define cross-reactivity and cross-blockade induced in naive mice and humans following GI.3 NoV infections.

Human HBGAs are an essential factor in NoV host range and also serve as a strong selection of NoV evolution (Dai et al., 2017). All the three studied GI NoV in the present study can bind to A, B, AB, and O secretors and non-secretors with some variations in binding affinity. GI.2 showed a high binding affinity to A, but weak signals to other phenotypes. Comparatively, GI.3 exhibited high affinity to A, AB and O secretors, which was concordant with r645 strain (Shirato et al., 2008). Sequence analysis revealed that GI.2 and GI.3 shared highly conserved HBGA binding interface and surrounding areas, with only one amino acid substitution (Q354/K355/E355) at the surrounding area (Figure 2), The finding is consistent with previous studies as mutations in surrounding areas result in the affinity changes (Lindesmith et al., 2008; Debbink et al., 2013; Zhang et al., 2015). Compared to GI.3, GI.9 gained a high affinity to non-secretors, but lower affinities to A and AB, which could be explained by the two mutations at the binding interface and the surrounding area. To our knowledge, the binding feature of GI.9 is firstly characterized and it shows broad-spectrum affinity to all HBGA phenotypes, which partly explain GI.9 as the highest asymptomatic NoV infection in Changsha Bay farm sites (Wang et al., 2018).

Consistent with immunotype clustering (Parra et al., 2017), GI.3 and GI.9 strains were closely related using phylogenetic analysis of P domain amino acid sequences but were less related to GI.2 strains. Following NoV infection with the GI.3, sera in convalescent phase exhibited the strongest IgG response against homotypic GI.3 with comparable titers against GI.9 and lower titer against GI.2 with no statistical difference. The seroconversion rate and strength among the three strains for the specific IgG titer and increased folds, which was also supported with the hyperimmune mice seroresponse in our study and another study (Lindesmith et al., 2010).

Parra analyses (Parra et al., 2017) suggested that most re-infections occurred with NoVs genotypes from different immunotypes but rarely within immunotype strains, suggesting complete or partial protection within an immunotype, but little to no protection between different immunotypes (Malm et al., 2014; Thorne et al., 2018). Our findings on murine serum systems showed serum of GI.2 failed to exert the blocking effect against GI.3 and GI.9. Meanwhile, the serum of GI.3 and GI.9 showed low blocking effect against all heterologous strains. These findings were consistent with a previous GI study that revealed that GI.1 VLP-immunized mouse did not produce GI.3 serum blocks, but GI.3-immunized mouse serum blocks heterologous GI.1 VLP (Malm et al., 2015).

Since human NoVs cannot effectively infect mice, they do not have pre-existing antibodies which hinder serological comparisons. Therefore, paired sera collected from NoV outbreak will afford valuable information for defining the correlation between protection and the magnitudes of pre-existing antibodies, as well as homologous and heterologous immune response. Of the selected GI NoVs immunotypes, we observed strong blockade in homologous strain, moderate blockade in intra-immunotypes but weak blockade between inter-immunotypes in humans following GI.3 NoV infections, which provided further support for Parra’s hypothesis. In the re-infection data of Parra’s study (Parra et al., 2017), they found a notable exception in immunotype G (GII.4 and GII.20), all eight re-infections were constraint within GII.4, which could be explained by re-infection with different variants of GII.4 viruses. Another exception was also observed in immunotype C (GI.3, GI.7, GI.8, and GI.9), which could be understandable as moderate blockade observed in intra-immunotype C in our finding. Meanwhile, weak blockade between inter-immunotypes B and C were observed, which was consistent with previous studies in GII NoVs (Dai et al., 2017; Karangwa et al., 2017), due to limited immune memory to former infection.

In this study, 66.7% of the infected cases showed relatively high IgG titers between 1,000 and 8,000 at the acute phase, However, only 9.52% of individuals showed low blockade antibody titers at 50 against GI.3 variant. The result revealed a low specific blockade antibody titer at the beginning of the outbreak, indicating a high proportion of susceptible individuals in the general population supports virus spread.

Previous studies showed that symptomatic individuals have greater immune system activation compared to asymptomatic individuals suggesting symptoms may be immune-mediated in norovirus infection (Newman et al., 2016). Notably, no differences of serological responses of IgG and blockade antibody between symptomatic patients and asymptomatic patients were found. A possible reason for this discrepancy may be due to the strong young soldier patients involved in our study who had strong immune system activation and response without symptoms.

In conclusion, the study represented an investigation of a gastroenteritis outbreak related to GI.3 NoV. Broad-spectrum HBGA binding profiles and evolutionary binding affinity were observed in GI.2, GI.3, and GI.9. A low specific blockade antibody in the population generated the pool of susceptible individuals and supported virus spread in the outbreak. We found strong blockade immune response in homologous strain, moderate blockade in intra-immunotypes but weak blockade between inter-immunotypes among humans following GI.3 NoV infections. These findings further support the immunotypes grouping and will be valuable for optimizing NoV vaccine design.

## Data Availability Statement

The datasets presented in this study can be found in online repositories. The names of the repository/repositories and accession number(s) can be found below: https://www.ncbi.nlm.nih.gov/genbank/, MT860989 and https://www.ncbi.nlm.nih.gov/genbank/, MT862216.

## Ethics Statement

The studies involving human participants were reviewed and approved by Ethics Committee, Nanfang Hospital, Southern Medical University. The patients/participants provided their written informed consent to participate in this study. The animal study was reviewed and approved by Southern Medical University Experimental Animal Ethics Committee.

## Author Contributions

DX and JC finished the experiment together and wrote the first draft and contributed equally to this work. Y-CD and X-FZ corresponding joint authors, designed and managed this study, provided project funding, and revised the manuscript. JY and FP made contributions in analyzing and interpreting data. MK, JY, and FP participated in the writing. LW helped develop methods and data analysis. MQ and YH participated in the design of the study. All authors contributed to the article and approved the submitted version.

## Conflict of Interest

The authors declare that the research was conducted in the absence of any commercial or financial relationships that could be construed as a potential conflict of interest.
